# Transcriptome Sequencing Reveals Key Genes in Three Early Phases of Osteogenic, Adipogenic, and Chondrogenic Differentiation of Bone Marrow Mesenchymal Stem Cells in Rats

**DOI:** 10.3389/fmolb.2021.782054

**Published:** 2022-02-11

**Authors:** Fanxiao Liu, Jun Dong, Peng Zhang, Dongsheng Zhou, Qingyu Zhang

**Affiliations:** Department of Orthopaedics, Shandong Provincial Hospital Affiliated to Shandong First Medical University, Jinan, China

**Keywords:** bone marrow mesenchymal stem cells, high-throughput sequencing, osteogenic differentiation, chondrogenic differentiation, adipogenic differentiation, differentially expressed genes

## Abstract

Bone mesenchymal stem cells (BMSCs) of multi-directional differentiation and reproductive activity are attractive candidates for bone and cartilage repair. However, the molecular mechanisms underlying the early phase of osteogenesis, adipogenesis, and chondrogenesis of BMSCs are still far from understood. In the current study, BMSCs are isolated from rats, and the gene expressions during the initiation of differentiation (phase I), lineage acquisition (phase II), and early lineage progression (phase III) of three-directional differentiation of BMSCs were detected by using high-throughput sequencing. Then, 356, 540, and 299 differentially expressed genes (DEGs) were identified in phases I, II, and III of osteogenesis, respectively. The numbers are 507, 287, and 428 for adipogenesis, respectively, and 412, 336, and 513 for chondrogenesis, respectively. Time-dependent expression patterns of genes were also validated during three-directional differentiation in BMSCs. Hub genes including *Ccna2*, *Cdc20*, and *Il6* may act as common participants in initiating osteogenesis, adipogenesis, and chondrogenesis. *Mex3b*, *Sertad1*, and *Hopx* showed an enhanced expression throughout three early phases during the osteogenic differentiation but no significant change in other two-directional differentiation. A similar pattern of *Dtx4* and *Ibsp* expression occurred in adipogenesis and chondrogenesis, respectively. Our findings will help understand the underlying mechanism determining the differentiation fate of BMSCs and provide theoretical support for the clinical treatment of osteoporosis, osteoarthritis, and other age-related bone diseases.

## Introduction

The microenvironment within the bone marrow cavity consists of both stromal lineage and hematopoietic lineage. Mesenchymal stem cells (MSCs) are discovered by Friedenstein et al. from stromal tissues of the bone marrow (BM) more than 50 years ago and have the potential to give rise to multiple mesodermal progenies such as osteocytes, adipocytes, and chondrocytes (Arthur and Gronthos, 2020; Jiang et al., 2021; Johnson et al., 2021; Saeedi et al., 2021). Meanwhile, bone MSCs (BMSCs) possess immune-modulatory and anti-inflammatory abilities (Li et al., 2012). Therefore, cell therapy with BMSCs has been proposed as an eligible paradigm for treating trauma, bone defects, and bone metabolic disorders (e.g., osteonecrosis and osteonecrosis) (Galderisi et al., 2021; Mabuchi et al., 2021). However, to date, clinical trials of MSC transplantation for osteoporosis predominantly focused on applying autologous cells, and no results have been reported (Jiang et al., 2021). Many unsolved problems and challenges still exist (Galderisi et al., 2021; Mabuchi et al., 2021; Saeedi et al., 2021). The first one is the patient selection and source of stem cells. A small number of MSCs can be readily obtained for autologous cellular transplantation, and therefore, extensive expansion may be required for therapeutic utility (Tamama et al., 2006). Meanwhile, there is a lack of homogenous criteria for isolating and cultivating MSCs and standardized procedures for transplantation. Last but not least, the differentiation fate of transplanted BMSCs is not controllable *in vivo*.

Adipogenesis, osteogenesis, and chondrogenesis of BMSCs are closely related. The specific differentiation direction is highly regulated by biological, physical, and chemical incentives (Hu et al., 2018). As the most studied terminally differentiated cell of BMSCs, the osteocyte is a mechanosensory commander maintaining homeostasis of the bone by osteoclasts and osteoblasts (Kim and Adachi, 2021). An imbalance resulting BMSCs toward a higher rate of adipogenesis is detrimental to bone health and associates with loss of bone mass and development of musculoskeletal diseases. Bone marrow adipocytes (BMAs) account for 50–70% of the marrow volume in adults (Piotrowska and Tarnowski, 2021). Besides acting as an energy reservoir, this kind of adipose tissue has been proved to possess paracrine, endocrine, and immunomodulatory functions (Sulston and Cawthorn, 2016). It, therefore, is critical for the homeostasis of the bone environment. Chondrocytes can be found in the articular surface of adults, which has poor regenerative capacity. Autologous chondrocyte transplantation is appealing for treating osteoarthritis, but the requirement for biopsies of chondrocytes from a healthy area of the cartilage cap is an evident disadvantage (Boeuf and Richter, 2010). Multiple key genes, non-coding RNAs (miRNA, lncRNA, and circRNA), and signaling pathways deciding the fate of BMSCs have been proposed to act at different time points during differentiation. For instance, the early stage of adipogenic differentiation is marked by augmentation of AMP cyclic production and phosphorylation of CREB (Chen et al., 2016). While during early osteogenesis, there is an upregulation of hedgehog proteins, Wnt/β-catenin signaling, BMPs, endocrine hormones, growth factors, and transcription factors (TFs) represented by RUNX2 (Pierce et al., 2019). SOX9 is pivotal for chondrogenesis by securing chondrocyte lineage commitment, promoting cell survival, and transcriptionally activating the genes for many cartilage-specific structural components (Lefebvre and Dvir Ginzberg, 2017).

Lineage commitment refers to the process of differentiation in which BMSCs lose their multipotency. During this phase, changes consistent with their respective lineage occur in the transcriptional profile, cellular metabolism, and morphology of BMSCs (van de Peppel et al., 2017). A primary question about the differentiation process is at what moment during the differentiation course MSCs become committed to a specific phenotype. Robert and his colleagues suggested that key genes related to osteogenesis were not significantly modulated in the first 24 h of osteogenesis of MSCs, and conversely, in MSCs treated with the adipogenic medium for 24 h, TFs related to adipogenesis and genes associated with lipid metabolism were already differentially expressed (Robert et al., 2020). van de Pepple et al. (2017) divided the early stage (4 d) of osteogenic and adipogenic lineage commitment of human BMSCs into three distinct phases: initiation of differentiation (0–3 h), lineage acquisition (6–24 h), and early lineage progression (48–96 h). The first phase is characterized by expression changes of many TFs and during the second phase, more than 50% of regulated genes are direct targets of the crucial TFs identified in the first phase. In the third phase, the number of differentially expressed genes (DEGs) is stable, indicating that differentiating cells have reached a stable phenotype. However, the author only applied BMSCs of human sources and key genes during chondrogenesis which are not investigated.

Many studies suggested that BMSCs from four species (human, pig, rat, and guinea pig) functioned across species barriers and act as promising sources for xenograft (Li et al., 2012). Currently, a change at the transcriptional level of rat BMSCs during the lineage-commitment phase of differentiation, especially chondrogenesis, remains unclear. In this study, using high-throughput sequencing data and multiple bioinformatics methods, we attempted to reveal key genes deciding osteogenic, adipogenic, and chondrogenic differentiation of rat BMSCs in early phases and analyze the enriched functions and their correlations. Meanwhile, potentially related microRNAs (miRNAs) and signaling pathways were predicted to elucidate underlying regulatory networks. These findings may help to characterize regulatory programs controlling the early stages of lineage commitment of rat BMSCs, to develop novel therapies for age- and pathology-related musculoskeletal diseases and to protect transplanted xenogeneic MSCs from immune detection and enable their long-term survival.

## Materials and Methods

**FIGURE 1:**
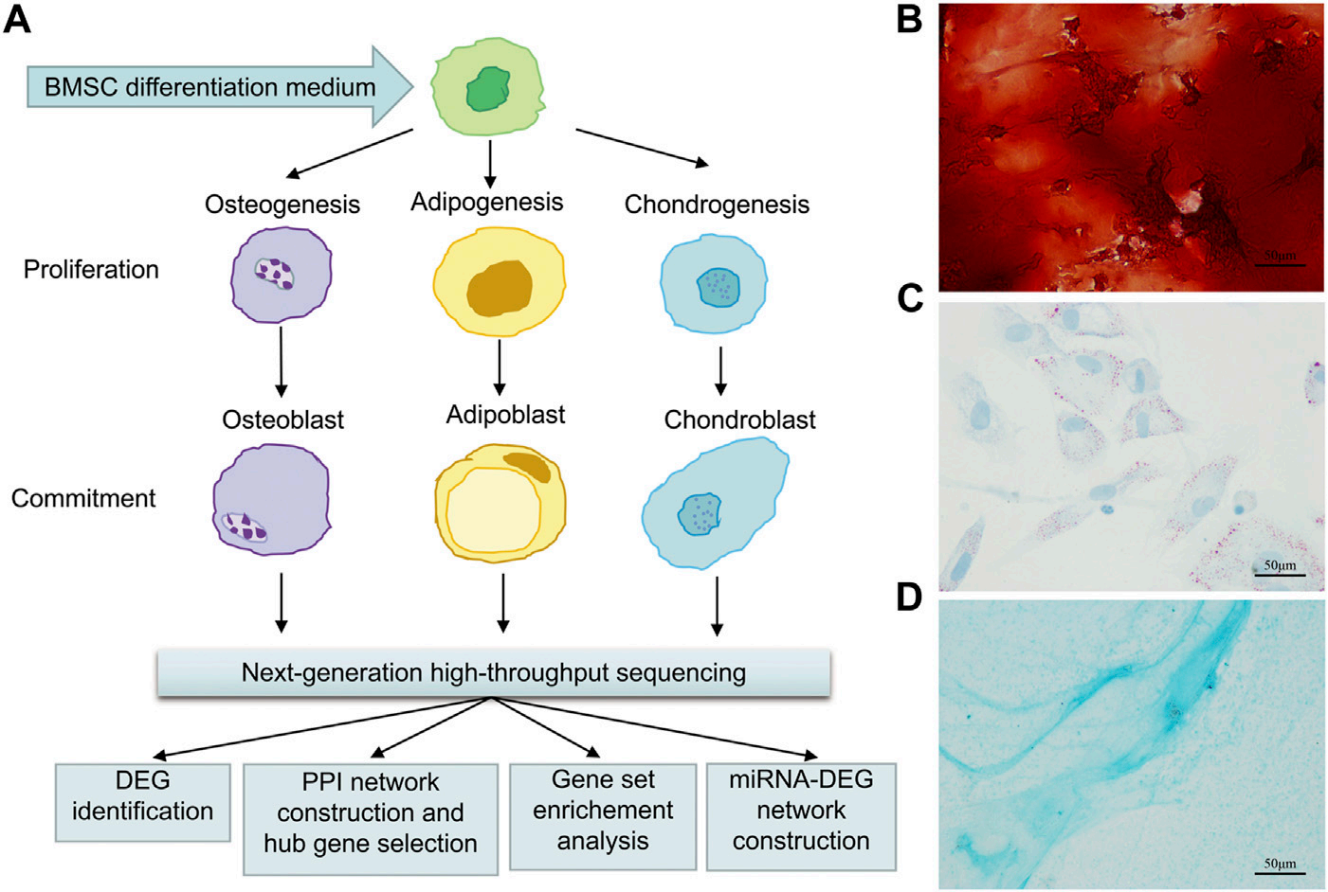
Flow chart of osteogenic, adipogenic, and chondrogenic differentiation of rat BMSCs, data gathering and analysis **(A)**, and histochemical staining of calcium **(B)** with Alizarin red or adipocyte **(C)** with oil red O or cartilage **(D)** with Alcian blue staining after 21 days of osteogenic or adipogenic or chondrogenic differentiation. Scale bars, 50 μm.

This study was approved by the Animal Ethics Committee of Shandong Provincial Hospital affiliated to Shandong First Medical University. The experimental design is depicted in Figure 1A.

### BMSC Isolation and Induced Differentiation

BMSCs were extracted from femurs and tibia of three female Sprague–Dawley (SD) rats about 3 weeks old by washing the marrow cavity using the high glucose Dulbecco’s Modified Eagle medium (DMEM) (Gibco, Rockville, MD, United States). The cells were cultured in the DMEM supplemented with 10% fetal bovine serum (FBS) and placed in an incubator with a temperature of 37°C, 5% CO_2_ concentration, and 95% relative humidity. BMSCs at passage 2 were seeded in 12-well cell culture plates. After 2 days of incubation (query), these cells were induced to give rise to osteoblasts by using the osteogenic differentiation medium (Promocell, Germany, C-28013), or to adipocytes using the adipogenic differentiation medium (Promocell, Germany, C-28016), or to chondrocytes using the chondrogenic differentiation medium (Promocell, C-28012). Differentiation media were replaced every 3–4 days.

### BMSCs Staining

For confirming the result of induced differentiation, Alizarin red staining, Oil Red O (ORO) staining, and Alcian blue staining assays were conducted, as previously described (Bruedigam et al., 2011; Gari et al., 2016).

### RNA Isolation

To obtain enough RNA for the gene expression profiling analyses, we pooled three individual cultures in TRIzol (Life Technologies). A total of three experimental samples per time point (3 time points, namely, 3, 12, and 72 h) per lineage (osteogenic, adipogenic, or chondrogenic) were used for gene expression profiling analyses. In addition to the control samples, there were a total of 30 samples.

### Next-Generation High-Throughput Sequencing and Identification of DEGs

The RNA quality assessment, mRNA library construction, and mRNA sequencing were performed using methods described in the previous study (Zhang et al., 2021). Based on the annotation information in the platform, the probe sets were transformed into the corresponding gene symbol. The mean value was calculated if multiple probes corresponded to the same gene symbol. The data were normalized using quantile normalization with Illumina in R software (Version 3.6.2). After data normalization, the DEGs were identified using the limma package (Version 3.42.2) in R software. Individual *p*-values were converted to adjusted *p*-values (adj. *p*. val) by false discovery rate correction of the Benjamini and Hochberg test. The threshold of |log_2_fold change (FC)| ≥ 1 with an adj. *p*. val <0.05 was used as the threshold for selecting DEGs. The volcano plot of the DEGs was drawn using the R ggplot2 package.

### Functional and Pathway Enrichment Analyses of DEGs

The DEGs were uploaded to an online biological information database, the Database for Annotation, Visualization, and Integrated Discovery (DAVID) version 6.8 Beta (https://david-d.ncifcrf.gov/) for gene ontology (GO) enrichment (http://www.geneontology.org/) and Kyoto Encyclopedia of Genes and Genomes (KEGG) pathway enrichment (https://www.genome.jp/kegg/pathway) analysis, which was visualized in the R ggplot2 package. The significance criterion was set at *p*. val <0.05.

### Protein–Protein Interaction Network Construction and Module Analysis

The protein–protein interaction (PPI) network of identified genes was constructed using the multiple protein online tool in the STRING database (Liu et al., 2021), a Search Tool for the Retrieval of Interacting Genes database (version 11.0b, http://string-db.org), and visualized by Cytoscape software (Version 3.6.2). The most significant modules in the PPI network were visualized using the molecular complex detection (MCODE) tool in Cytoscape, and the hub genes were selected by the cytoHubba plugin. The selection criteria were as follows: MCODE scores >5, node score cutoff = 0.2, degree cutoff = 2, max depth = 100, and k-score = 2.

### Key Gene Selection

The hub genes (top 10) in the PPI network were identified by using three methods, including maximal clique centrality (MCC), degree, and maximum neighborhood component (MNC) from the cytoHubba plugin in Cytoscape. The biological process analysis of the top 10 genes was performed and visualized using the Biological Networks Gene Ontology tool (BiNGO) (version 3.0.3) plugin in Cytoscape.

### DEG–miRNA Network Construction

After hub genes were selected, the DEG–miRNA pairs were identified using the miRDB database (http://mirdb.org/) and visualized in Cytoscape.

## Results

### Confirmation of the Three-Directional Differentiation of Rat BMSCs

On day 21 after induced differentiation, Alizarin red staining for calcium in the osteogenic differentiation group showed mineralization of the extracellular matrix (Figure 1B); meanwhile, intracellular lipid vesicles accumulated in the adipogenic group (Figure 1C), and cartilage formed in the chondrocyte differentiation group (Figure 1D). Taken together, these results confirmed that rat BMSCs differentiated in accordance with their expected multi-lineage potential.

### Data Normalization

**FIGURE 2:**
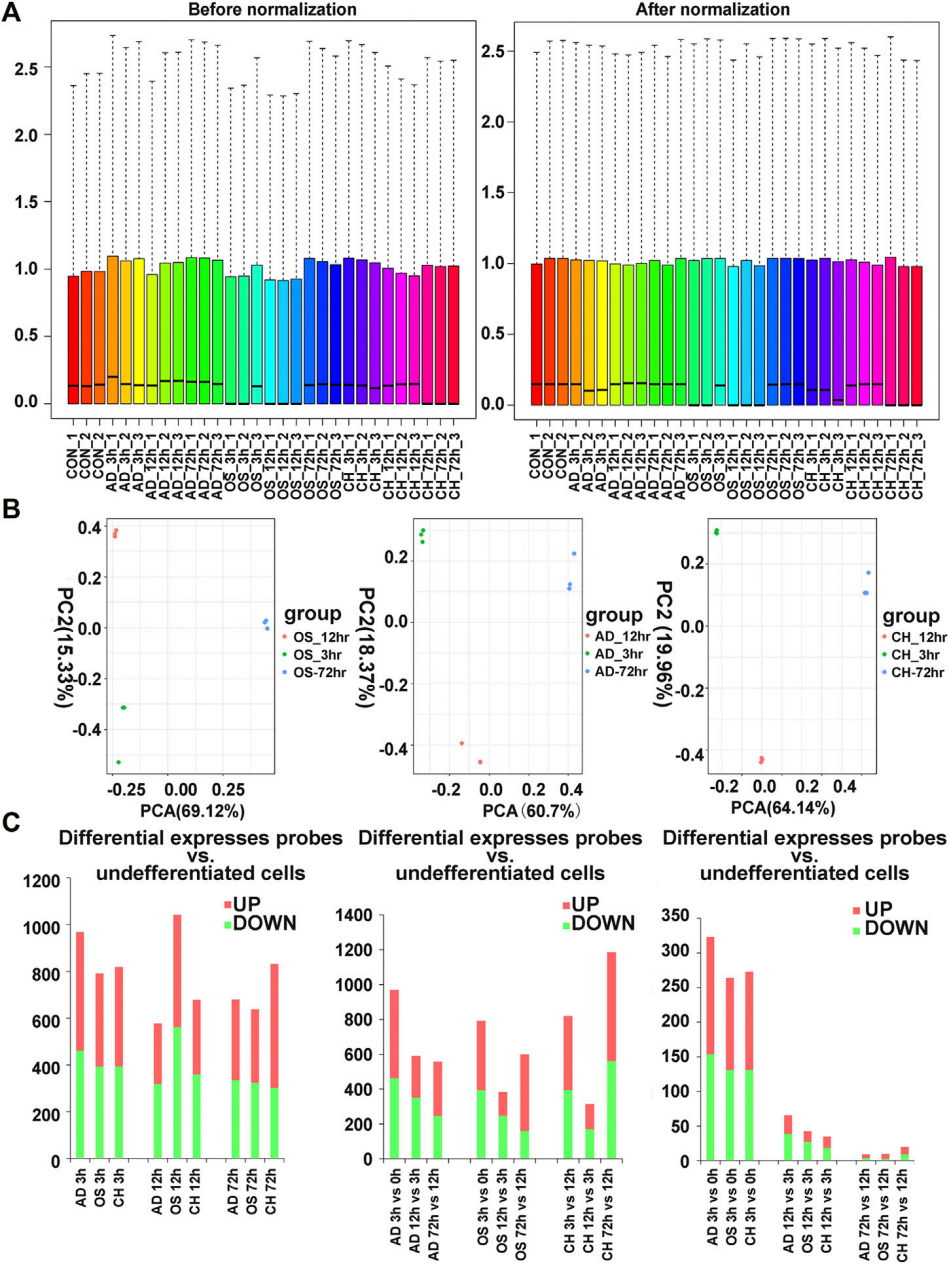
**(A)** Distribution of expression of 27 samples before and after normalization. **(B)** Distribution of expression of 27 samples involving principal component analysis (PCA) for confirming biological variability between different samples. **(C)** Dynamic transcriptional changes upon osteogenic, adipogenic, and chondrogenic differentiation of rat BMSCs with the number of significant differential genes relative to the previous time point (based on three independent experiments) and the number of differentially expressed genes per hour compared to the previous time point (based on three independent experiments). AD, adipogenesis; OS, osteogenesis; CH, chondrogenesis.

The box plot visualization of the gene expression of all samples (Figure 2A) was used to evaluate the data normalization and cross-comparability, which demonstrated that the black lines were almost in the same position after data normalization. Principal component analysis (PCA) confirmed the biological variability between different samples, indicating globally distinct expression profiles (Figure 2B).

### Identification of DEGs at Three Time Points

Analysis of gene expression dynamics during rat BMSC differentiation revealed that transcript levels changed significantly at 3 h upon induction of differentiation (Figure 2C). During induction of osteogenic differentiation, 792 genes (398 upregulated and 394 downregulated ones) were significantly different at 3 h, and this increased further to 1,042 (480 upregulated and 562 downregulated ones) at 12 h. The number of significantly modulated genes decreased to 638 (314 upregulated and 324 downregulated ones) at 72 h. During adipogenic differentiation, the number of DEGs was 969 at 3 h, 577 at 12 h, and 680 at 72 h. During chondrogenic differentiation, the number of DEGs was 819 at 3h, 678 at 12 h, and 832 at 72 h. Remarkably, at 3 h after differentiation, the number of upregulated DEGs was higher than the downregulated ones. Next, we calculated the number of differentially expressed DEGs at each time point compared with the preceding time point, and in three directions of differentiation, the number of DEGs that changed per hour decreased as time extended (Figure 2C).

**FIGURE 3:**
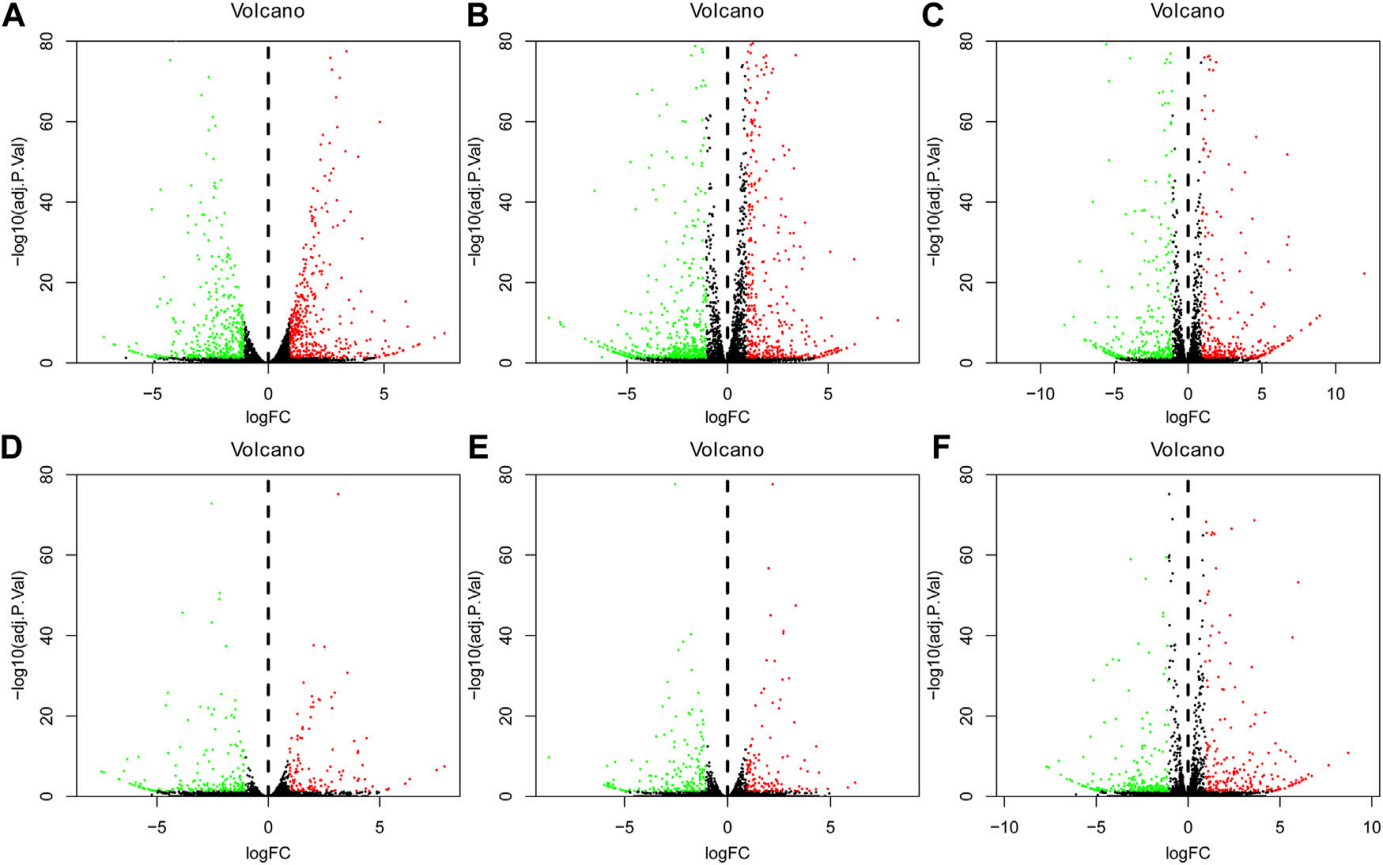
Volcano plot of differentially regulated genes during osteogenesis at 3 h, **(A)** 12 h, and **(B)** 72 h **(C)** Compared with undifferentiated cells (*t* = 0 h); Volcano plot of differentially regulated genes between every two lineages at 3 h after inductive differentiation (*t* = 0 h). **(D)** Comparison between osteogenesis and adipogenesis, **(E)** Comparison between osteogenesis and chondrogenesis, and **(F)** Comparison between chondrogenesis and adipogenesis. AD, adipogenesis; OS, osteogenesis; CH, chondrogenesis.

The volcano plot of DEGs at 3, 12, and 72 h of osteogenesis and the comparison results between every two lineages at 3 h after inductive differentiation are presented in Figure 3. Supplementary Figure S1 describes the visualized results of DEGs at three time points of adipogenic and chondrogenic differentiation.

### Identification of Specific DEGs at Three Time Points

**FIGURE 4:**
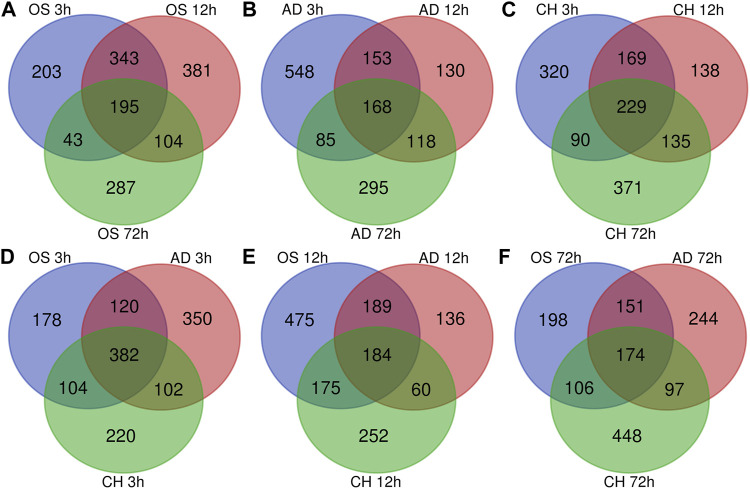
Venn Diagrams of differentially expressed genes during osteogenesis **(A)**, adipogenesis **(B)**, and chondrogenesis **(C)** at three time points compared with undifferentiated cells (t = 0 h). Venn Diagrams of differentially expressed genes at 3 h **(D)**, 12 h **(E)**, and 72 h **(F)** of three directions of differentiation compared with undifferentiated cells (t = 0 h). AD, adipogenesis; OS, osteogenesis; CH, chondrogenesis.

In osteogenesis of BMSCs, 203, 381, and 287 DEGs were identified to be differentially expressed only at 3, 12, and 72 h after inductive differentiation (Figure 4A). In adipogenesis of BMSCs, the numbers were 548, 130, and 295 (Figure 4B), and for chondrogenesis of BMSCs, the numbers were 320, 138, and 371 (Figure 4C). Notably, 195 (e.g., Mex3b and Sertad1), 168(e.g., Dtx4 and Il6), and 229 (e.g., Glra1 and Fdps) genes showed differentially expressed levels at all three time points in osteogenesis, adipogenesis, and chondrogenesis, respectively.

### Comparison of DEGs Between Different Lineages

At 3 h after induction of differentiation, it was noticed that there were 178, 350, and 220 DEGs only differentially expressed in osteogenesis, adipogenesis, and chondrogenesis, respectively (Figure 4D). At 12 h after inductive differentiation, the numbers were 475, 136, and 252 (Figure 4E), and at 72 h, the numbers were 198, 244, and 448 (Figure 4F). Notably, 382 (e.g., *Hmg20a* and *Mmp24*), 184 (e.g., *Galnt2* and *Cxcl6*), and 174 (e.g., *Ccnd2* and *Sgcd*) genes showed differential expression at all three directions of differentiation at 3, 12, and 72 h after inductive differentiation, respectively.

### Functional Enrichment Analysis of DEGs

**FIGURE 5:**
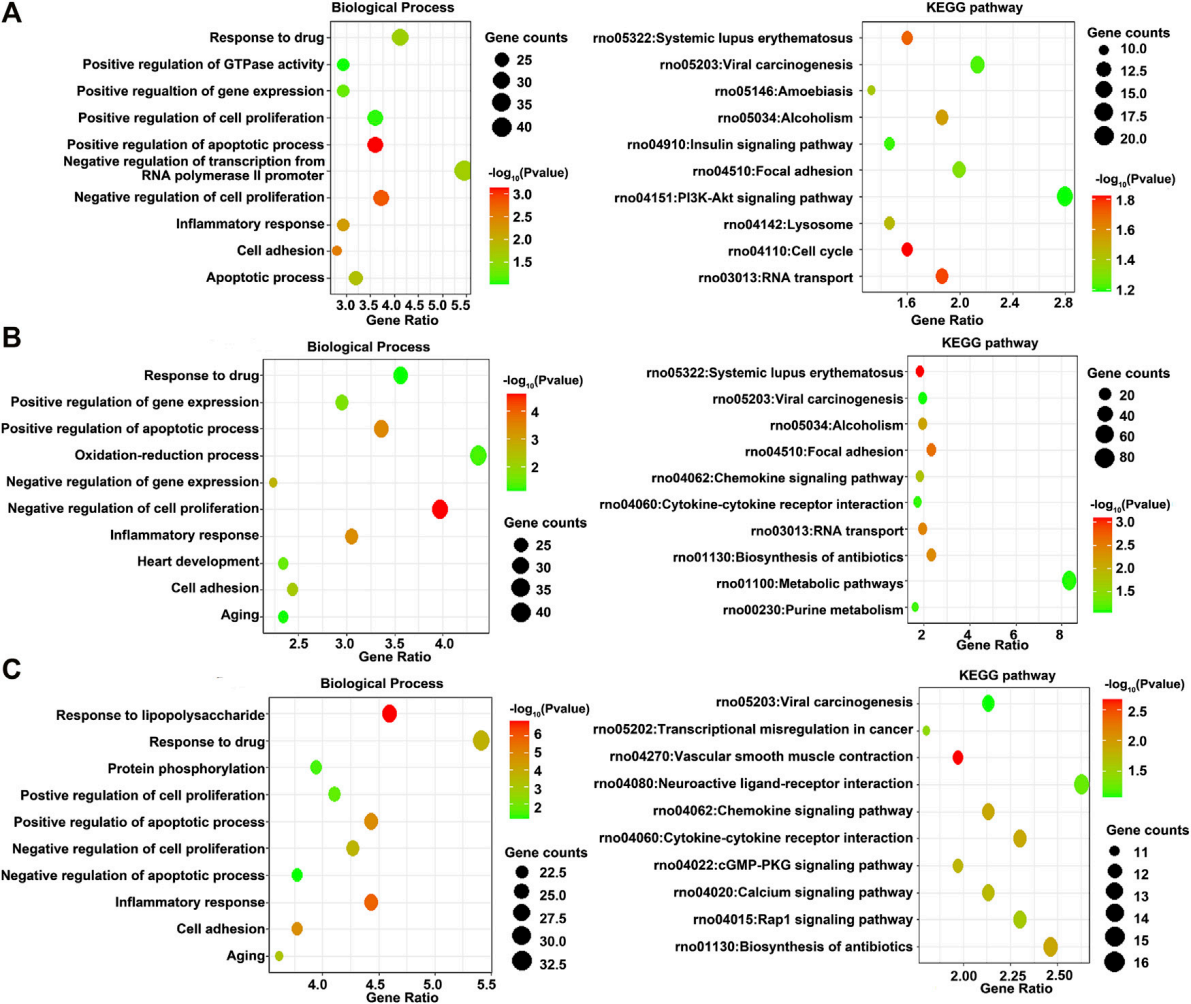
GO including the biological process and KEGG enrichment analysis of differentially expressed genes at 3 h, **(A)** 12 h, and **(B)** 72 h **(C)** of osteogenesis compared with undifferentiated cells (*t* = 0 h).

GO and KEGG pathway enrichment analyses of differentially expressed DEGs at different time points of three directions of differentiation were performed to identify the most relevant biological processes (BPs), molecular functions (MFs), cellular components (CCs), and pathways. The top enriched terms of DEGs in BP, CC, MF, and KEGG at 3 h after osteogenesis are presented in Figure 5A and Supplementary Figure S2A. The DEGs at 3 h of osteogenesis were significantly enriched in the “PI3K-Akt signaling pathway” and BP terms such as “negative regulation of transcription from RNA polymerase II promoter” and “response to drug.” The enriched terms at 12 and 72 h after osteogenesis are presented in Figures 5B,C and Supplementary Figures S2B,C, while results of the other two directions of differentiation are presented in Supplementary Figures S3, S4.

**FIGURE 6:**
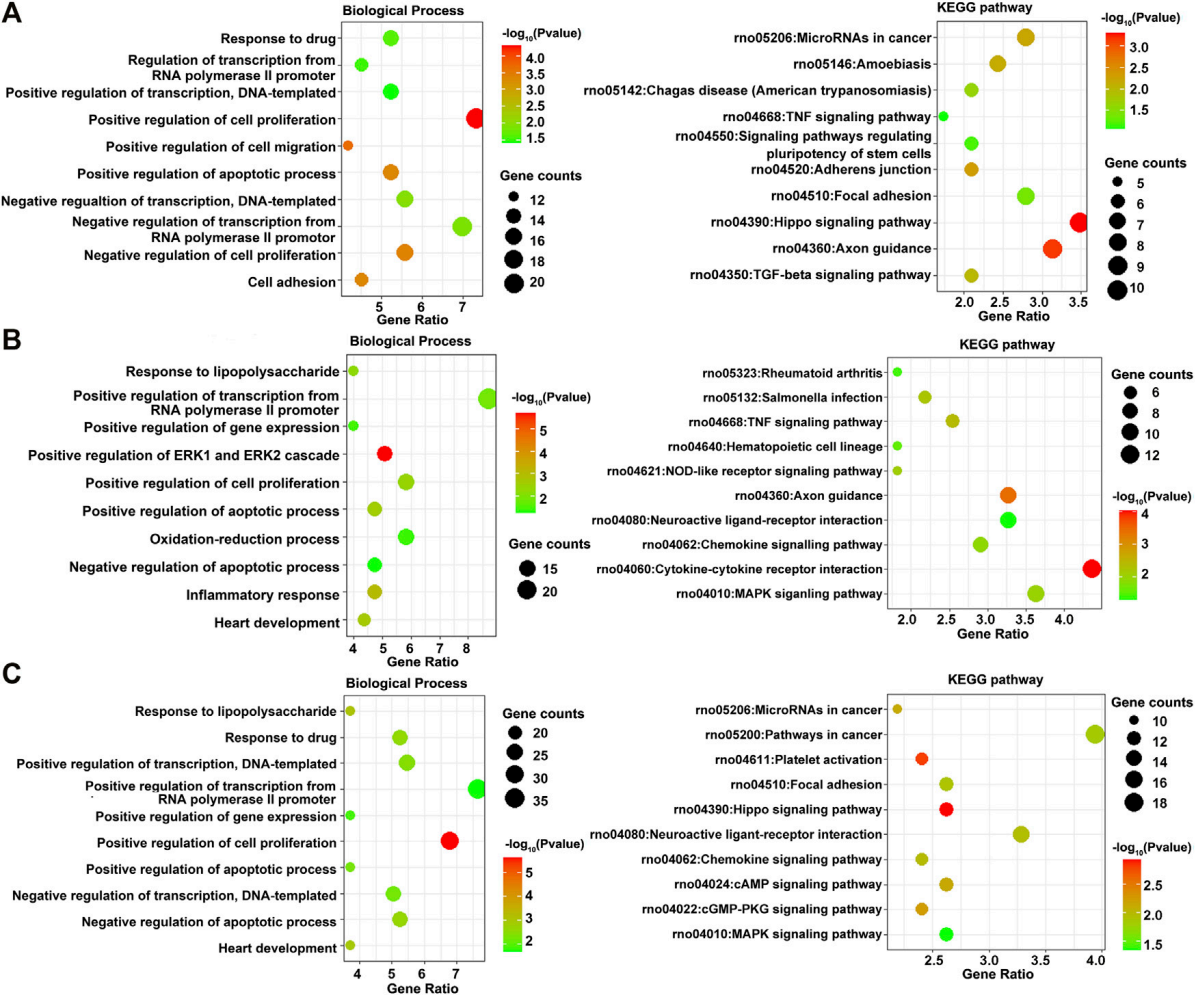
GO including the biological process and KEGG enrichment analysis of differentially expressed genes between every two lineages at 3 h after inductive differentiation (*t* = 0 h). **(A)** Comparison between osteogenesis and adipogenesis, **(B)** Comparison between osteogenesis and chondrogenesis, and **(C)** Comparison between chondrogenesis and adipogenesis.

The DEGs between osteogenesis and adipogenesis at 3 h after inductive differentiation were also significantly enriched in BP terms such as “positive regulation of cell migration” and “negative regulation of transcription from RNA polymerase II promoter.” Results of functional enrichment analysis of the DEGs between every two lineage commitment at 3 h of inductive differentiation are presented in Figure 6 and Supplementary Figure S5.

### PPI Network Construction and Module Analysis

**FIGURE 7:**
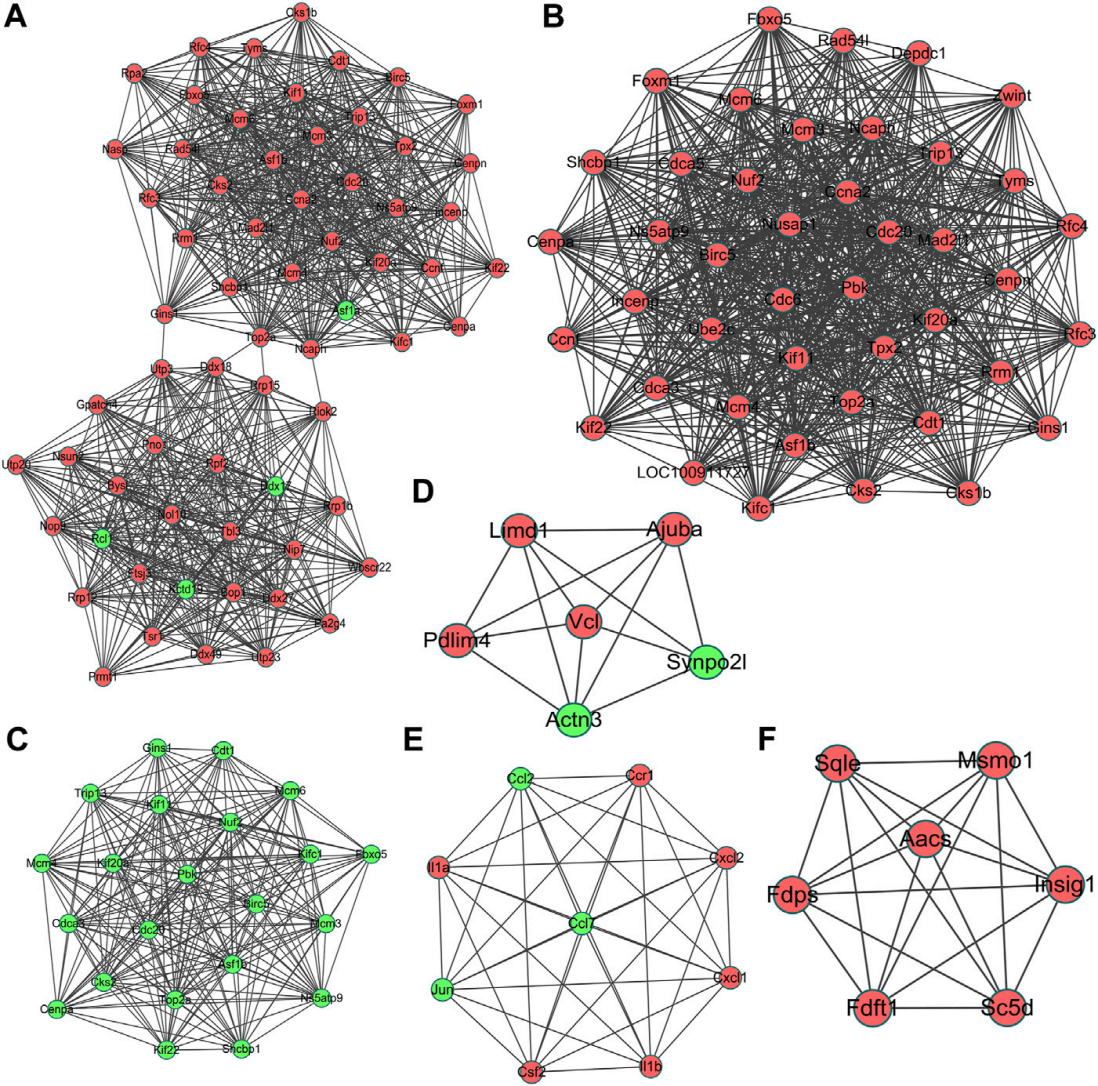
Most significant module in protein–protein interaction (PPI) network analysis of differentially expressed genes at 3 h, **(A)** 12 h, and **(B),** and 72 h **(C)** of osteogenesis compared with undifferentiated cells (*t* = 0 h); The most significant module of differentially regulated genes between osteogenesis and adipogenesis, **(D)** between osteogenesis and chondrogenesis, and **(E)** between chondrogenesis and adipogenesis **(F)** at 3 h after inductive differentiation (*t* = 0 h). Red indicates upregulated genes, and green indicates the downregulated genes.

**FIGURE 8:**
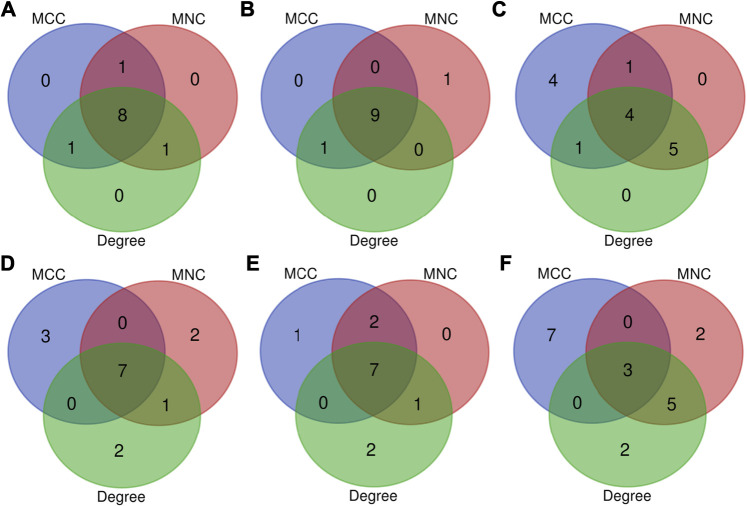
Top 10 key genes at 3 h, **(A)** 12 h, and **(B)** 72 h **(C)** of osteogenesis compared with undifferentiated cells (*t* = 0 h); The top 10 key genes identified from comparisons between osteogenesis and adipogenesis, **(D)** between osteogenesis and chondrogenesis, and **(E)** between chondrogenesis and adipogenesis **(F)** at 3 h after inductive differentiation (*t* = 0 h), which were selected based on the three most commonly used classification methods (MCC, MNC, and Degree) in cytoHubba.

**FIGURE 9:**
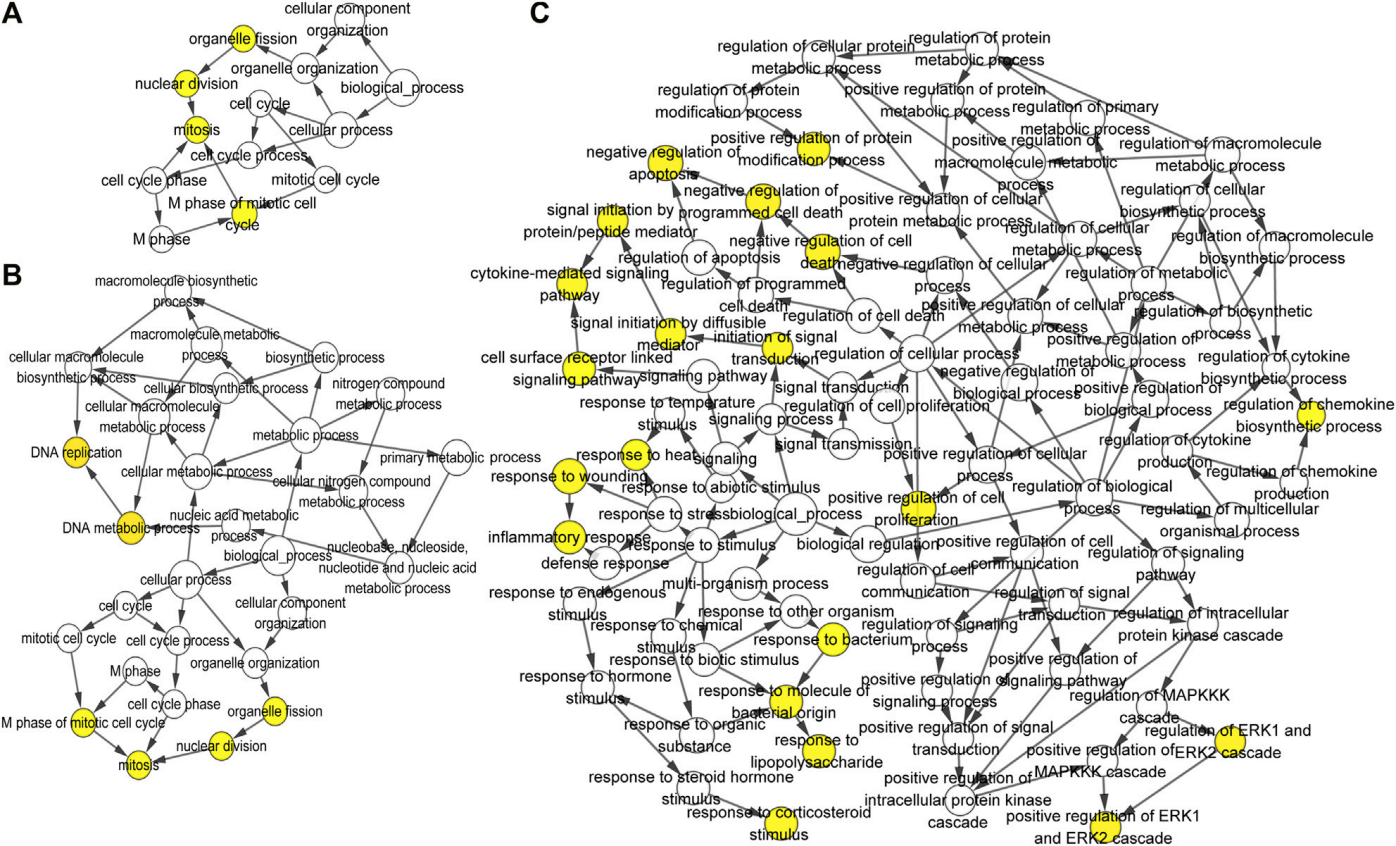
Biological process analysis of the top 10 differentially expressed genes was constructed using BiNGO at 3 h, **(A)** 12 h, and **(B)** 72 h **(C)** of osteogenesis compared with undifferentiated cells (*t* = 0 h). The color depth of nodes refers to the corrected *p*-value of ontologies. The size of nodes refers to the number of genes that are involved in the ontologies. *p* < 0.001 was considered statistically significant.

The most significant module of DEGs at 3 h of osteogenesis consisted of 65 nodes and 907 edges, with the Rrp15 gene being identified to have a relatively high connectivity degree (Figure 7A). The most significant modules at 12 and 72 h after osteogenesis are presented in Figures 7B,C, respectively. Meanwhile, the most significant modules of the other two-directional differentiation at three time points are presented in Supplementary Figure S6. The most significant module about interactions of DEGs between osteogenesis and adipogenesis at 3 h after inductive differentiation consisted of 7 nodes and 14 edges, with the *Vcl* gene being identified to have a relatively high connectivity degree (Figure 7D). The most significant modules regarding interactions of DEGs between osteogenesis and chondrogenesis and between adipogenesis and chondrogenesis at 3 h after inductive differentiation are presented in Figures 7E,F. Hub genes were selected by CytoHubba. The top 10 hub genes, which were selected based on the three most commonly used classification methods in cytoHubba, are presented in Figure 8 and Supplementary Figure S7. The top 10 hub genes selected by degree in different comparisons are presented in Table 1. The fold change (FC) and adj. *p*. val of the top 10 hub genes at 3, 12, and 72 h of osteogenesis compared with undifferentiated cells are presented in Table 2. The biological process analysis of the top 10 hub genes was performed and visualized using BiNGO in Cytoscape, as shown in Figure 9 and Supplementary Figures S8, S9.

**TABLE 1:** List of the top 10 hub genes selected by degree methods in cytoHubba.

Comparisons	Hub genes
OS3h vs BMSCs	Ccna2, Cdc20, Il6, Mad2l1, Top2a, Kif11, Mcm4, Asf1b, Rrm1, Pa2g4
OS12 h vs BMSCs	Il6, Mad2l1, Top2a, Ccna2, Cdc20, Mcm6, Mcm3, Kif11, Mcm4, Cdc6
OS72 h vs BMSCs	Il6, Cd44, Il1b, Ccl2, Kif11, Notch1, Asf1b, Cdc20, Mcm3, Pbk
AD3h vs BMSCs	Il6, Ccna2, Cdc20, Kif11, Top2a, Mad2l1, Gsk3b, Mcm6, Asf1b, Mcm3
AD12 h vs BMSCs	Ccna2, Mad2l1, Mcm3, Top2a, Mcm6, Kif11, Cdc20, Rrm1, Mcm4, Asf1b
AD72 h vs BMSCs	Il6, Notch1, Igf1, Il1b, Kif11, Cdc20, Asf1b, Kif20a, Top2a, Rhoc
CH3h vs BMSCs	Il6, Mad2l1, Top2a, Cdc20, Kif11, Ccna2, Mcm3, Sspo, Mcm6, Mcm4
CH12 h vs BMSCs	Il6, Jun, Notch1, Lep, Mmp2, Acta1, Pik3r1, Csf2, Ldlr, Gja1
CH72 h vs BMSCs	Il6, Rac1, Il1b, Acta1, Ccl2, Vcl, Ctnna1, Sox9, Actn2, Actg2
OS3h vs AD3h	Il6, Jun, Notch1, Pik3r1, Sox9, Vcl, Ctgf, Nck1, Rhoq, Gnai1
OS3h vs CH3h	Il6, Jun, Notch1, Sox9, Vcl, Rnd1, Ccl2, Ctgf, Notch2, Lpl
CH3h vs AD3h	Jun, Csf2, Sox9, Ccl2, Rasa1, Il1b, Epha2, Cxcl1, Il1a, Rnd1

OS, osteogenic differentiation; CH, chondrogenic differentiation; AD, adipogenic differentiation.

**TABLE 2:** The fold change (FC) and adj. *p*. val of the top 10 hub genes at 3, 12, and 72 h of osteogenesis compared with undifferentiated cells.

3 h			12 h			72 h		
Gene	log_2_FC	adj. *p*. val	Gene	log_2_FC	adj. *p*. val	Gene	log_2_FC	adj. *p*. val
Asf1b	2.07865	<0.01	Kif11	2.83993	<0.01	Il1b	−6.1996	<0.01
Ccna2	2.52671	<0.01	Mcm4	2.40732	<0.01	Asf1b	−2.4827	<0.01
Cdc20	2.35907	<0.01	Mcm6	3.31951	<0.01	Cd44	−1.1223	<0.01
Il6	−4.6024	<0.01	Mcm3	3.30175	<0.01	Cdc20	−2.2716	<0.01
Kif11	1.67841	<0.01	Cdc20	2.7624	<0.01	Ccl2	−3.2291	<0.01
Mad2l1	1.56818	0.0128	Il6	−6.5453	<0.01	Kif11	−2.833	<0.01
Mcm4	1.13145	<0.01	Top2a	3.34705	<0.01	Notch1	1.86226	<0.01
Pa2g4	1.31558	<0.01	Mad2l1	2.81097	<0.01	Pbk	−5.2452	<0.01
Rrm1	1.53775	<0.01	Ccna2	3.14156	<0.01	Il6	−6.3788	<0.01
Top2a	2.4447	<0.01	Cdc6	3.9125	0.0111	Mcm3	−1.8955	<0.01

### Construction of DEG-miRNA

**FIGURE 10:**
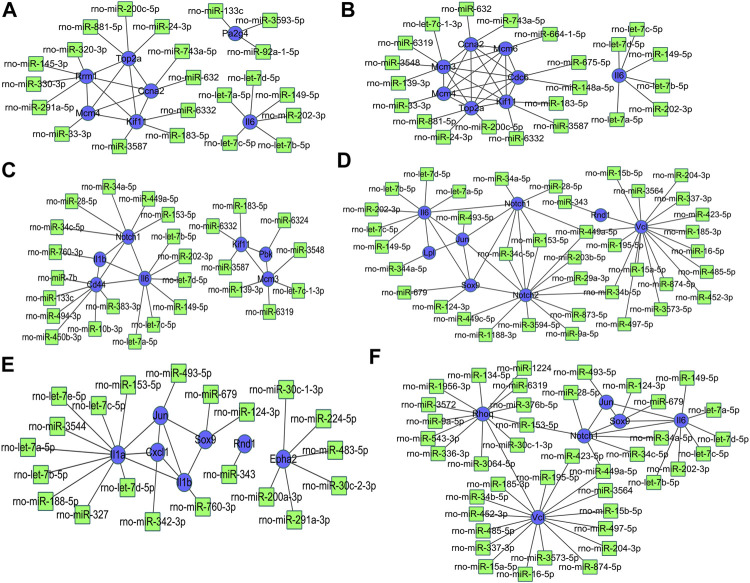
Regulatory network of DEG-miRNA was obtained from the miRDB database using the DEGs at 3 h, **(A)** 12 h, and **(B)** 72 h **(C)** in osteogenesis compared with undifferentiated cells (*t* = 0 h), and the DEGs identified from comparisons between osteogenesis and adipogenesis, **(D)** between osteogenesis and chondrogenesis, and **(E)** between chondrogenesis and adipogenesis **(F)** at 3 h after inductive differentiation (*t* = 0 h). Blue indicates genes, and green indicates targeted miRNA. DEG, differentially expressed gene; miRNA, microRNA.

The miRNA-DEG pairs were identified through network analysis of 10 DEGs using the miRDB databases. At 3 h of osteogenesis, a total of 32 associations between 22 miRNAs and 7 DEGs were identified, and then, the network was visualized in Cytoscape (Figure 10A). As is shown, *Rrm1* interacts with four miRNAs (rno-miR-320-3p, rno-miR-145-3p, rno-miR-330-3p, and rno-miR-291a-5p) and four genes (*Top2a*, *Ccna2*, *Mcm4*, and *Kif11*). The DEG-miRNA networks regarding osteogenesis at 12 and 72 h are presented in Figures 10B,C. The DEG-miRNA networks about comparisons between every two lineage commitment at 3 h after inductive differentiation are presented in Figures 10D–F. The DEG-miRNA networks regarding adipogenesis and chondrogenesis at 3, 12, and 72 h after differentiation are presented in Supplementary Figure S10.

## Discussion

There were no previous studies paying special attention to the lineage commitment of rat BMSCs (Arthur and Gronthos, 2020; Zhu et al., 2020). In the current investigation, three time points (3, 12, and 72 h) after inductive differentiation were chosen to represent the three phases of early lineage commitment of rat BMSCs. Next-generation high-throughput sequencing was adapted to generate potential key genes. At the first time point (3 h), gene expression analyses revealed many DEGs in all three directions of differentiation. The first phase of early lineage commitment represents the initiation stage of the differentiation program. It is characterized by expression changes of many transcription factors that regulate the lineage commitment and set the multipotential stem cells toward a stable phenotype (van de Peppel et al., 2017). As for the second phase, differentiating lineages begin to deviate. In this study, most genes related to osteogenesis were modulated later than those related to adipogenesis, which confirmed the finding of the study conducted by Robert and his colleagues (Robert et al., 2020). In the third stage, the differentiating cells reach a stable phenotype (van de Peppel et al., 2017).

Integrated analysis of differential gene expression at 3 h of osteogenesis indicated that the top 10 DEGs (*Ccna2*, *Cdc20*, *Il6*, *Mad2l1*, *Top2a*, *Kif11*, *Mcm4*, *Asf1b*, *Rrm1*, and *Pa2g4*) in the PPI network were the hub genes; only one gene (*Il6*) showed a decreasing trend compared to the control group. Similarly, at 12 h of osteogenesis, nine (*Mad2l1*, *Top2a*, *Ccna2*, *Cdc20*, *Mcm6*, *Mcm3*, *Kif11*, *Mcm4*, and *Cdc6*) of the identified top 10 hub genes in the PPI network were upregulated except *Il6*. This trend was reversed at 72 h of osteogenesis as it has been shown that at this moment, nine (*Il6*, *Cd44*, *Il1b*, *Ccl2*, *Kif11*, *Asf1b*, *Cdc20*, *Mcm3*, and *Pbk*) of the top 10 DEGs were downregulated except Notch1. For adipogenesis, eight (*Ccna2*, *Cdc20*, *Kif11*, *Top2a*, *Mad2l1*, *Mcm6*, *Asf1b*, and *Mcm3*) hub genes were upregulated and two (*Il6* and *Gsk3b*) were downregulated at 3 h of differentiation, while for chondrogenesis, only two (*Il6* and *Sspo*) hub genes were downregulated at this time point. In fact, there is rather a large overlap regarding hub genes identified at 3 h of osteogenesis, chondrogenesis, and adipogenesis. Three genes (*Ccna2*, *Cdc20*, and *Il6*) were upregulated in all three groups, which suggested that most identified hub genes at this phase may not play independent roles in determining the lineage commitment of BMSCs but act as common participants in initiating differentiation. *Ccna2* is ubiquitously expressed and acts as an essential regulator of the cell division cycle at the G1/S transition and mitotic entry (Bendris et al., 2012; Loukil et al., 2014). *Cdc20* is a key regulator of ubiquitylation of FRET and could colocalize with *Ccna2*, LC3-B, p62 or lysosomes (Loukil et al., 2014). Il6 is a well-known pleiotropic cytokine and shows multiple functions such as the regulation of inflammation and immunity (Hunter and Jones, 2015). Deng et al. (2020) have demonstrated that the expression of *Il6* increased during adipogenesis of MSCs and was positively correlated with the Oil Red O quantification result.

Unique DEGs that occurred in only one lineage may play pivotal roles in determining the fate of BMSCs. Five genes (*LOC689899*, *Mex3b*, *Sertad1*, *LOC100362684*, and *Hopx*) were dysregulated in three time points of osteogenesis but showed non-significant changes during two other lineages (Supplementary Table S1). *Mex3B*, which is reportedly to induce apoptosis after cell stimulations such as heat, radiation, and reactive oxygen species, is downregulated in osteogenesis (Umehara et al., 2020). *Hopx* has been demonstrated to be a promoter of BMSC proliferation and an inhibitor of adipogenesis by suppressing adipogenic pathway–related genes such as *PLIN1*, *FABP4*, and *PLIN5* (Hng et al., 2020). *Dtx4* was exclusively upregulated in three time points during adipogenesis and was involved in cell development and cell differentiation (Wang et al., 2017). Wang et al. (2017) indicated that knockdown of this gene could inhibit adipogenesis through restraining C/EBPα and PPARγ and therefore arresting mitotic clonal expansion. Sixteen genes were upregulated through the early lineage of chondrogenesis. Among them, the expression of *Ibsp* is driven by RUNX3 and MEF2C, which appears more critical than RUNX2 for chondrogenesis (Dreher et al., 2020). It could be noticed that a lot of genes are exclusively dysregulated at the first phase of differentiation, and their effect for determining the fate of rat BMSCs needs to be further confirmed (Supplementary Table S1). A total of 207 unique DEGs were only identified at 3 h of adipogenesis, which suggested that key genes related to adipogenesis were significantly modulated earlier than osteogenesis.

Then, various bioinformatics analysis methods were used to reveal the enriched functional terms regarding identified DEGs. As a result, GO terms of 792 DEGs identified at 3 h of osteogenesis for biological process categories included “negative regulation of transcription from RNA polymerase II promoter” and “response to drug.” Furthermore, the KEGG pathway enrichment analysis revealed that these DEGs were predominantly associated with the “PI3K-Akt signaling pathway.” The DEGs in the “PI3K-Akt signaling pathway” were *Chrm2*, *Pdgfra*, *Ngfr*, *Col24a1*, *Lama1*, *Lama4*, *Flt4*, *Pik3r5*, *Rbl2*, *Il6*, *Ppp2r1b*, *Fgf9*, *Col5a1*, *Col5a2*, *Col4a3*, *Eif4ebp1*, *Tek*, *Ywhah*, *Ifnar1*, *Pck2*, and *Epha2*. As to 12 h of osteogenesis, the most involved BP terms are “oxidation-reduction process” and “negative regulation of cell proliferation,” which suggest that BMSCs start from proliferation to differentiation at this phase. While in this phase, the most enriched KEGG pathway is “metabolic pathways.” Similar changes occurred early in adipogenesis in comparison with osteogenesis. It can be noticed that at 3 h of adipogenesis, the most enriched BP terms are “oxidation-reduction process,” “positive regulation of cell proliferation,” “negative regulation of apoptotic process,” and “response to drug,” while the most involved KEGG pathways are “metabolic pathways.”

miRNAs have been demonstrated to be important regulatory factors in the differentiation of BMSCs by binding the 3′-untranslated region of targeted mRNAs (Zhou et al., 2020; Liu et al., 2021). Thus, we constructed a DEG-miRNA regulatory network to show the potential interactions among identified DEGs and RNAs during osteogenesis, adipogenesis, and chondrogenesis. In the networks of DEGs about comparisons between adipogenesis and osteogenesis at 3h, rno-miR-449-5p may play an important role because it connects with three key genes. This miRNA, along with rno-miR-153-5p, also occurred in the network about comparisons between osteogenesis and chondrogenesis. Rno-miR449a-5p are essential for both mitochondria metabolic dysfunction and phenotype transformation of pulmonary arterial smooth muscle cells (Zhang et al., 2019), but currently, there is no direct evidence to prove the role of this miRNA for the lineage commitment of rat BMSCs.

For the first time, the gene transcription of rat BMSCs during osteogenesis, adipogenesis, and chondrogenesis was systematically detected and analyzed. The current study provided directions and molecular targets for further investigations on the lineage commitment of rat BMSCs by high-throughput sequencing and rigorous bioinformatics analysis. However, it should be admitted that there were shortcomings that merit consideration. First, this is only a very fundamental research study using two-dimensional culture. The differentiation in the internal environment may possess different characteristics in comparison with the *in vitro* experiment. These results cannot completely represent the normal physiological processes. The role of predicted genes and miRNAs in the three-directional differentiation of BMSCs merits further investigation. Second, only three time points distributed so-called “three phases of lineage commitment” were selected in this study as representatives. More detailed results may be acquired if more time points during differentiation were selected. Third, the DEG-miRNA network was constructed by using the miRDB database. In the future, we will detect the expression of non-coding RNA using our samples and then construct a competing endogenous RNA network. Last but not least, because there is a large number of differentially expressed genes identified in the diverse comparisons, validation by Western blot assay or protein array was not available. In the future, specific molecules could be selected for in-depth investigation.

## Conclusion

These hub genes, functional terms, and predicted targeted genes identified in the current study may provide information for further clarification of the molecular mechanism underlying the lineage commitment of rat bone mesenchymal stem cells. Hub genes including *Ccna2*, *Cdc20*, and *Il6* may act as typical participants in initiating osteogenesis, adipogenesis, and chondrogenesis. *Mex3b*, *Sertad1*, and *Hopx* showed enhanced expression throughout three early phases during the osteogenic differentiation but no significant change in other two-directional differentiation. A similar pattern of *Dtx4* and *Ibsp* expressions occurred in adipogenesis and chondrogenesis, respectively. Additionally, rno-miR-449-5p is a potential regulator for the early lineage commitment of osteogenesis. Our findings will help understand the underlying mechanism determining the differentiation fate of BMSCs and provide theoretical support for the clinical treatment of osteoporosis, osteoarthritis, and other age-related bone diseases.

## Data Availability

The datasets presented in this study can be found in online repositories. The names of the repository/repositories and accession number(s) can be found below: https://www.ncbi.nlm.nih.gov/geo/, accession ID: GSE185140.
