# Supplementary figures and images for: Transcriptome Sequencing Reveals Key Genes in Three Early Phases of Osteogenic, Adipogenic, and Chondrogenic Differentiation of Bone Marrow Mesenchymal Stem Cells in Rats
Source: Front Mol Biosci. 2022 Feb 11;8:782054. doi: 10.3389/fmolb.2021.782054 (PMC8873985; doi:10.3389/fmolb.2021.782054)

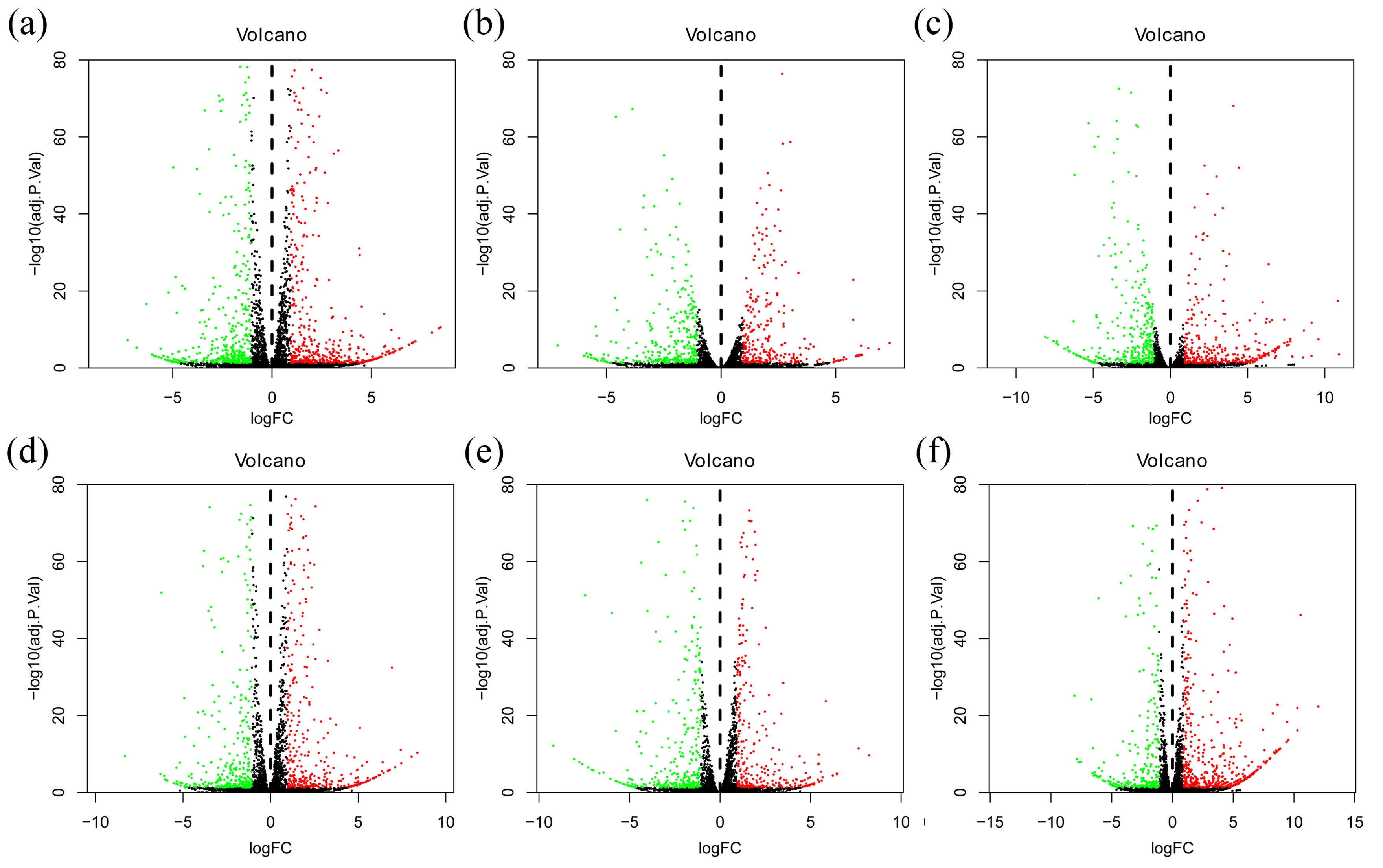

Supplement: Supplementary file 1 [file Image1.TIFF]

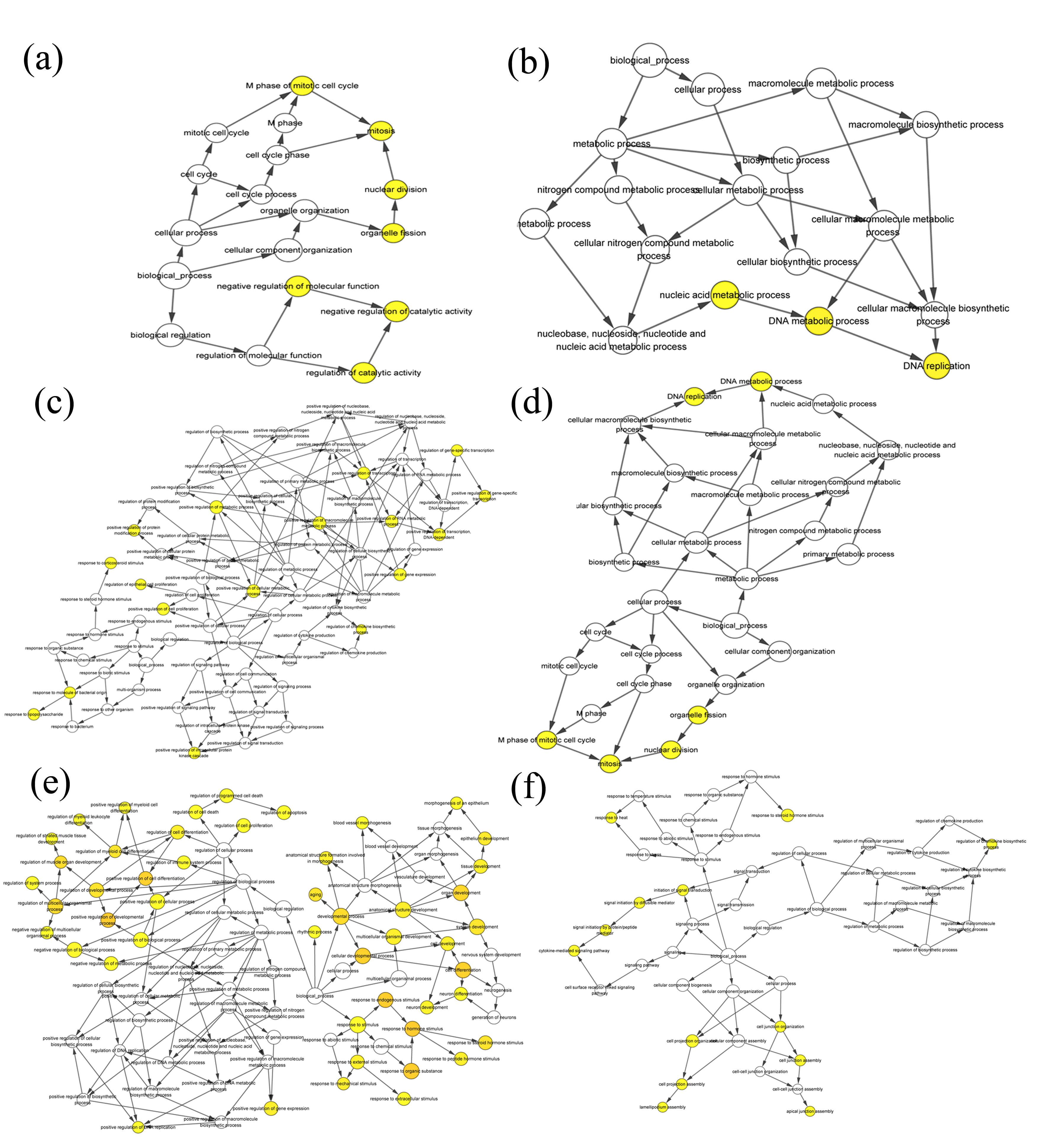

Supplement: Supplementary file 2 [file Image9.TIFF]

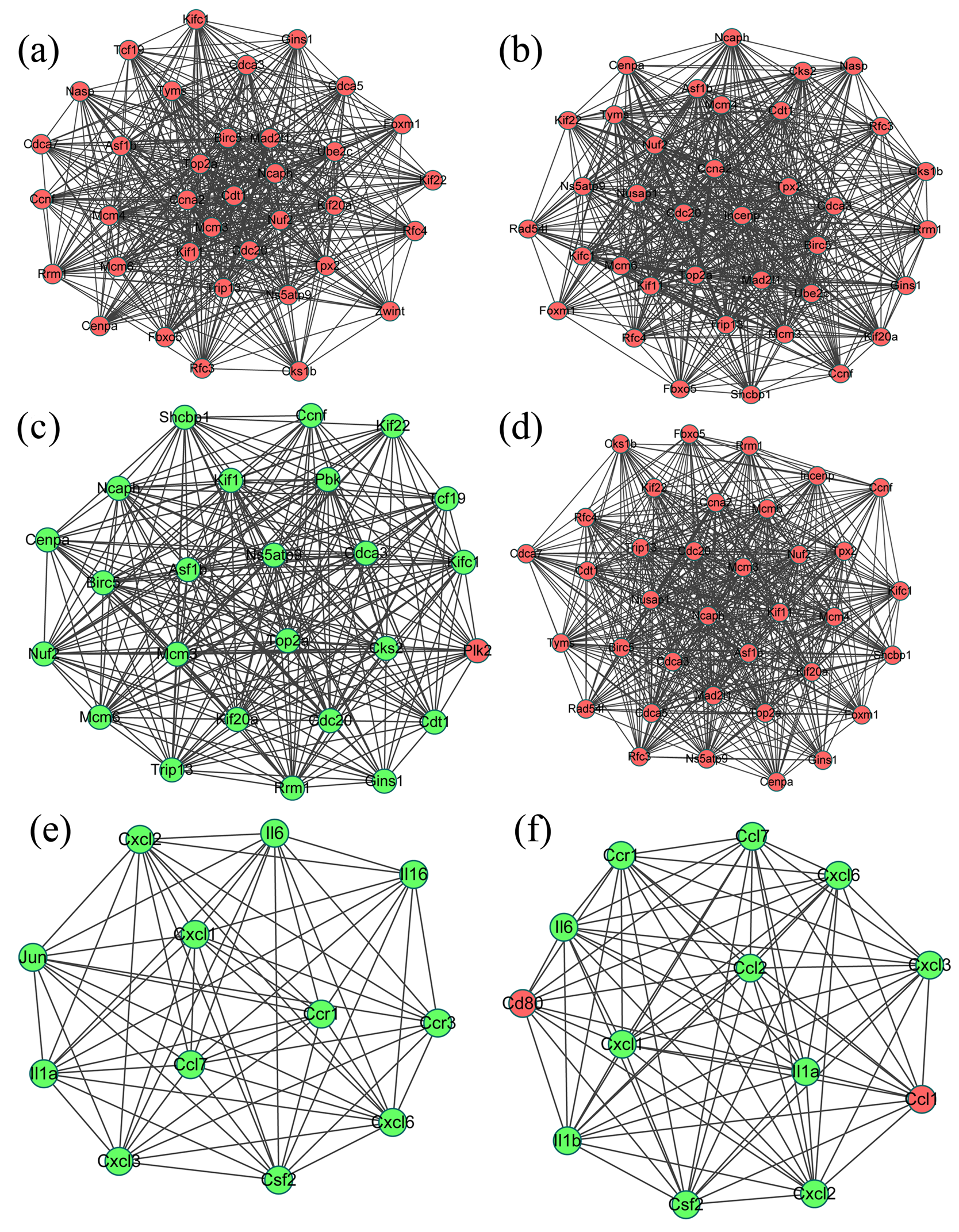

Supplement: Supplementary file 3 [file Image6.tif]

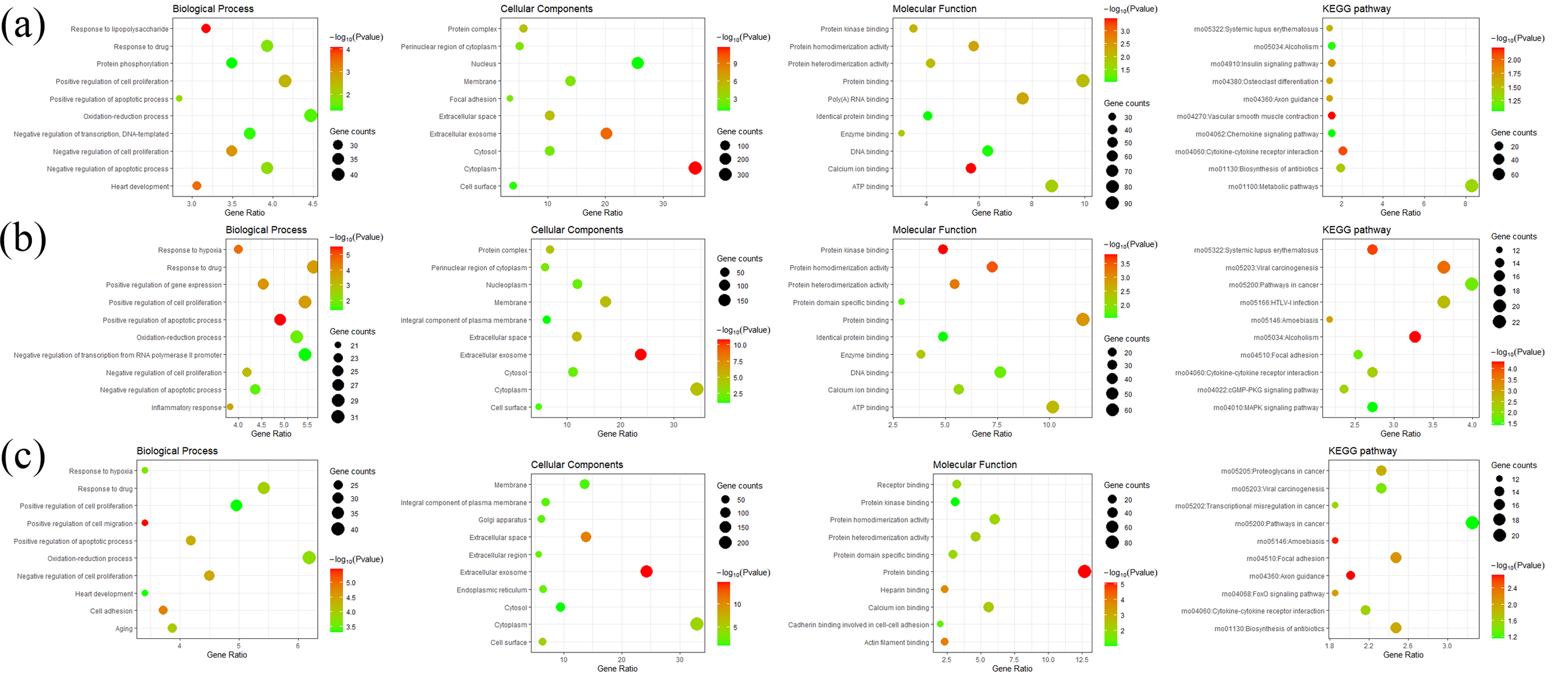

Supplement: Supplementary file 5 [file Image3.TIF]

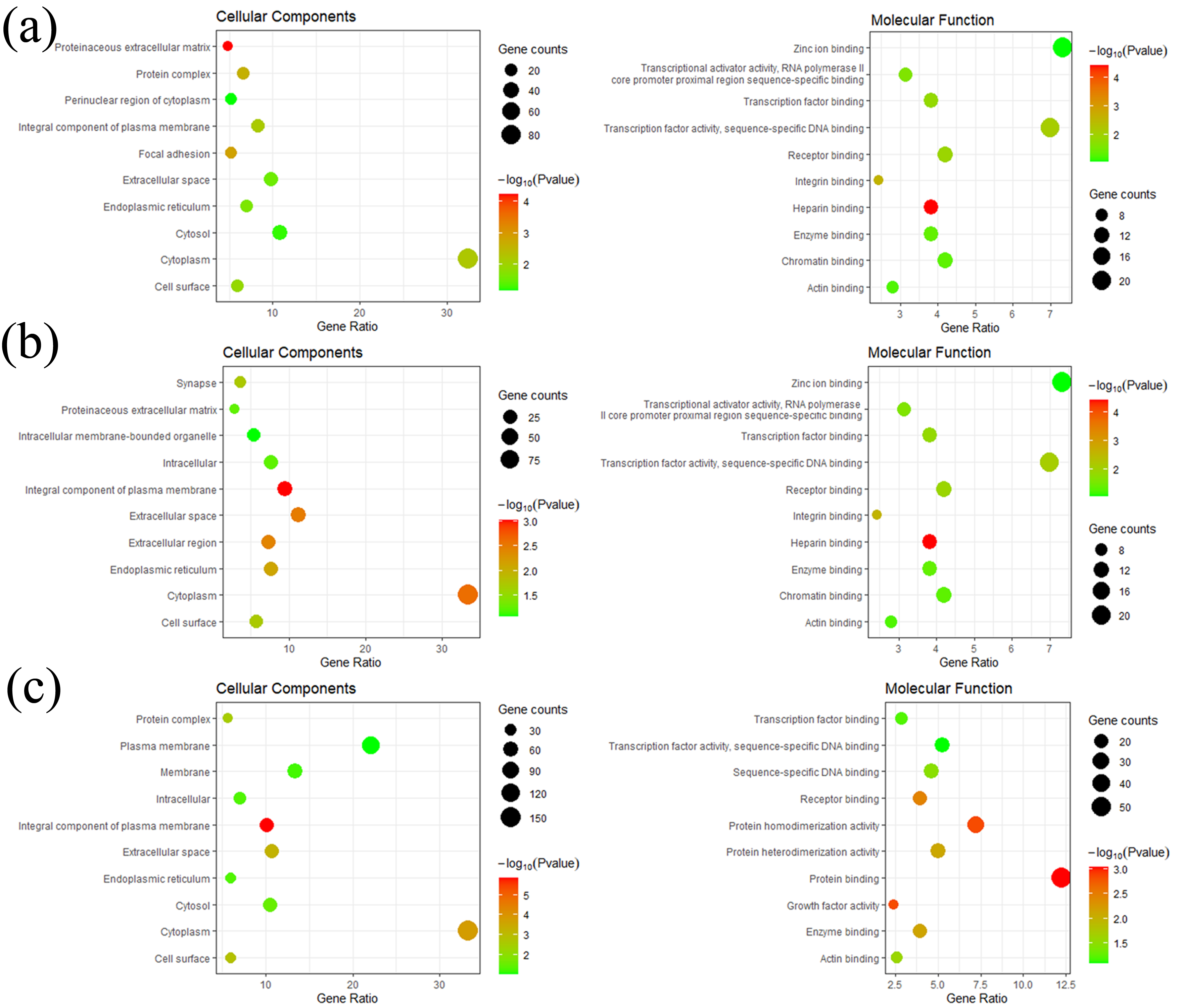

Supplement: Supplementary file 6 [file Image5.TIFF]

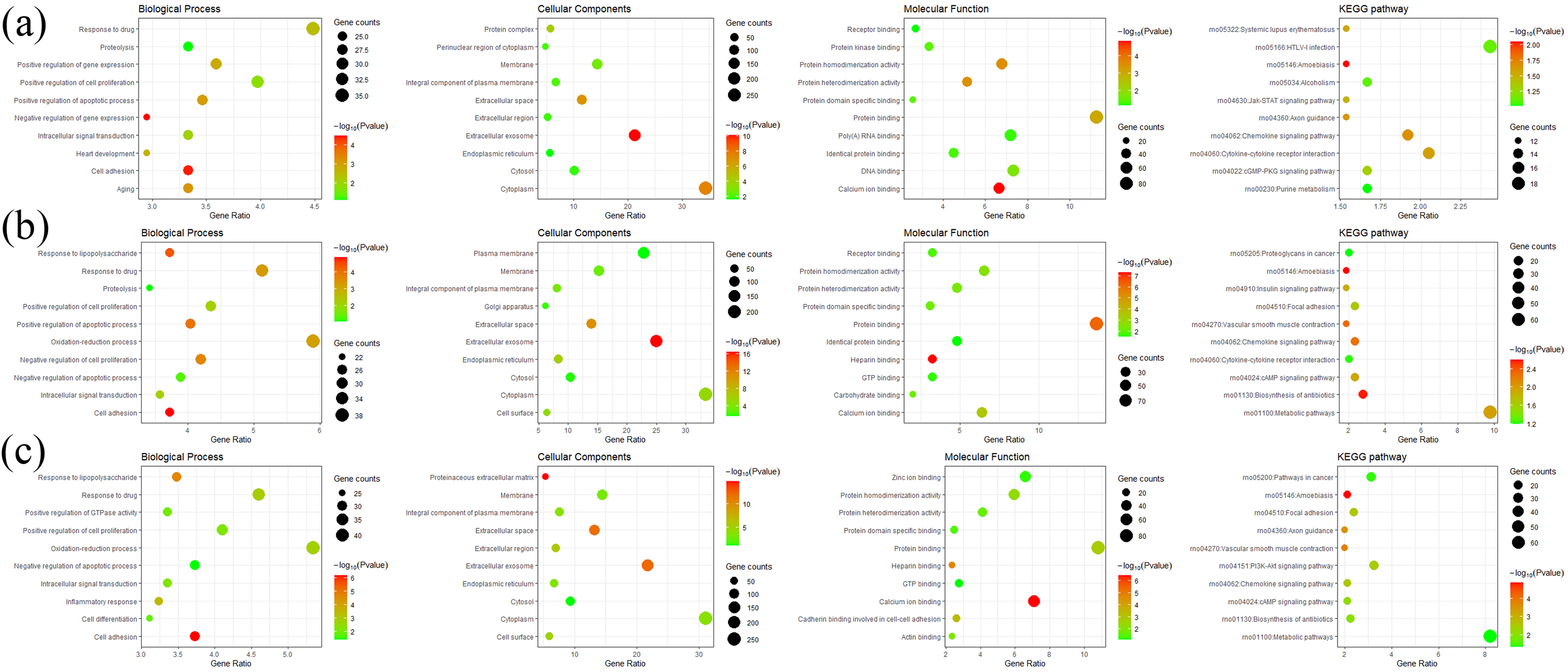

Supplement: Supplementary file 7 [file Image4.TIF]

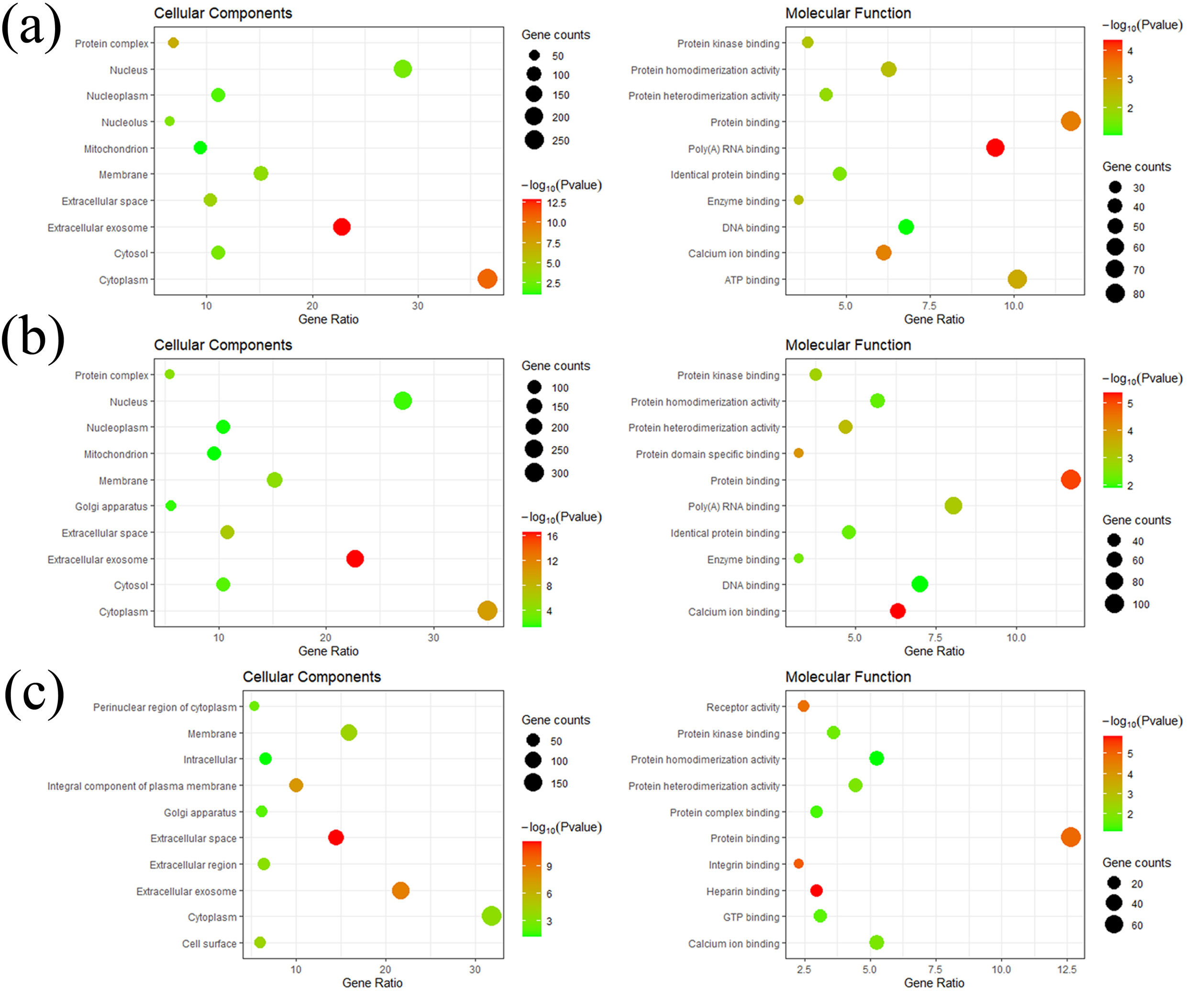

Supplement: Supplementary file 8 [file Image2.TIF]

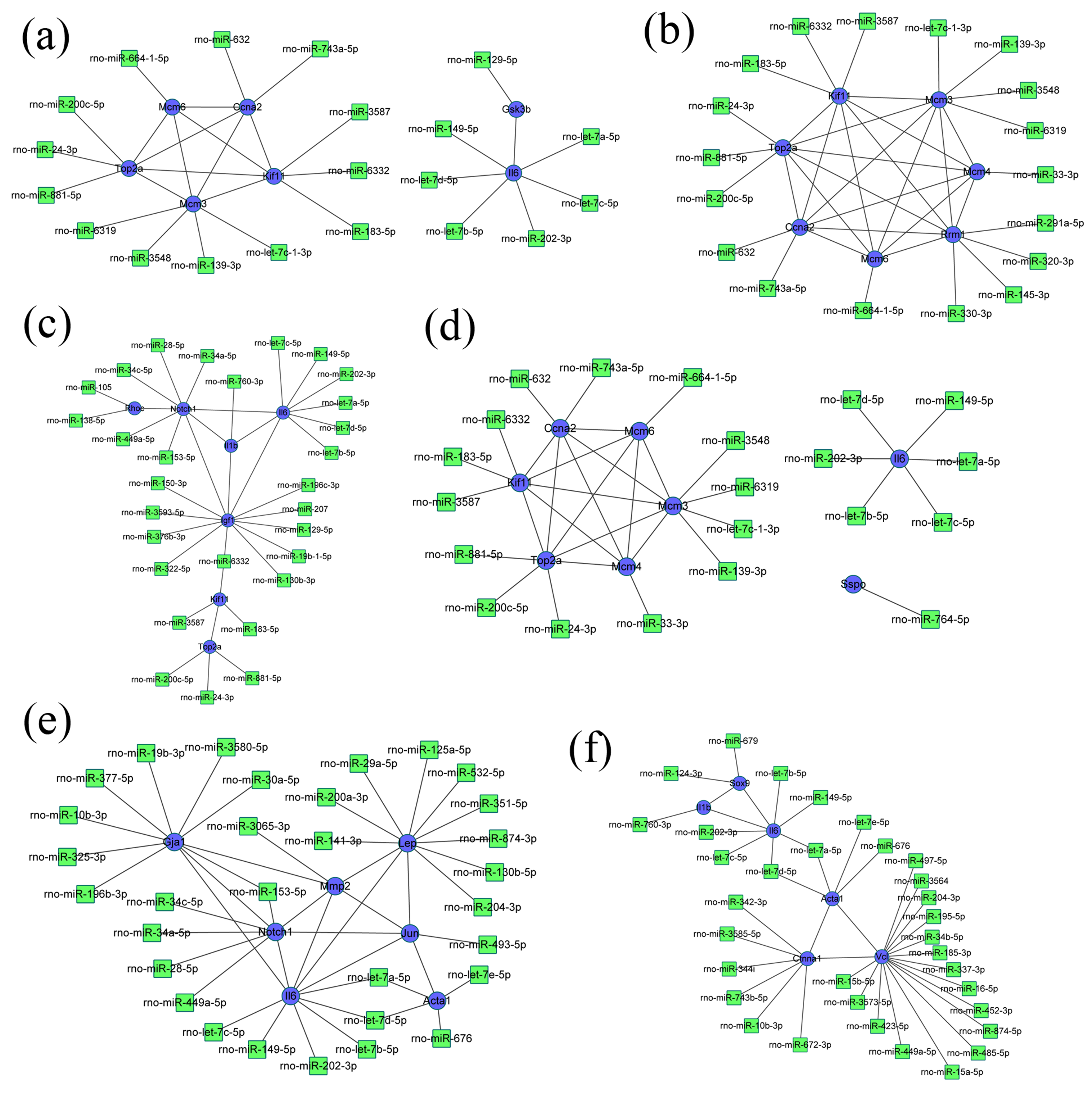

Supplement: Supplementary file 9 [file Image10.TIFF]

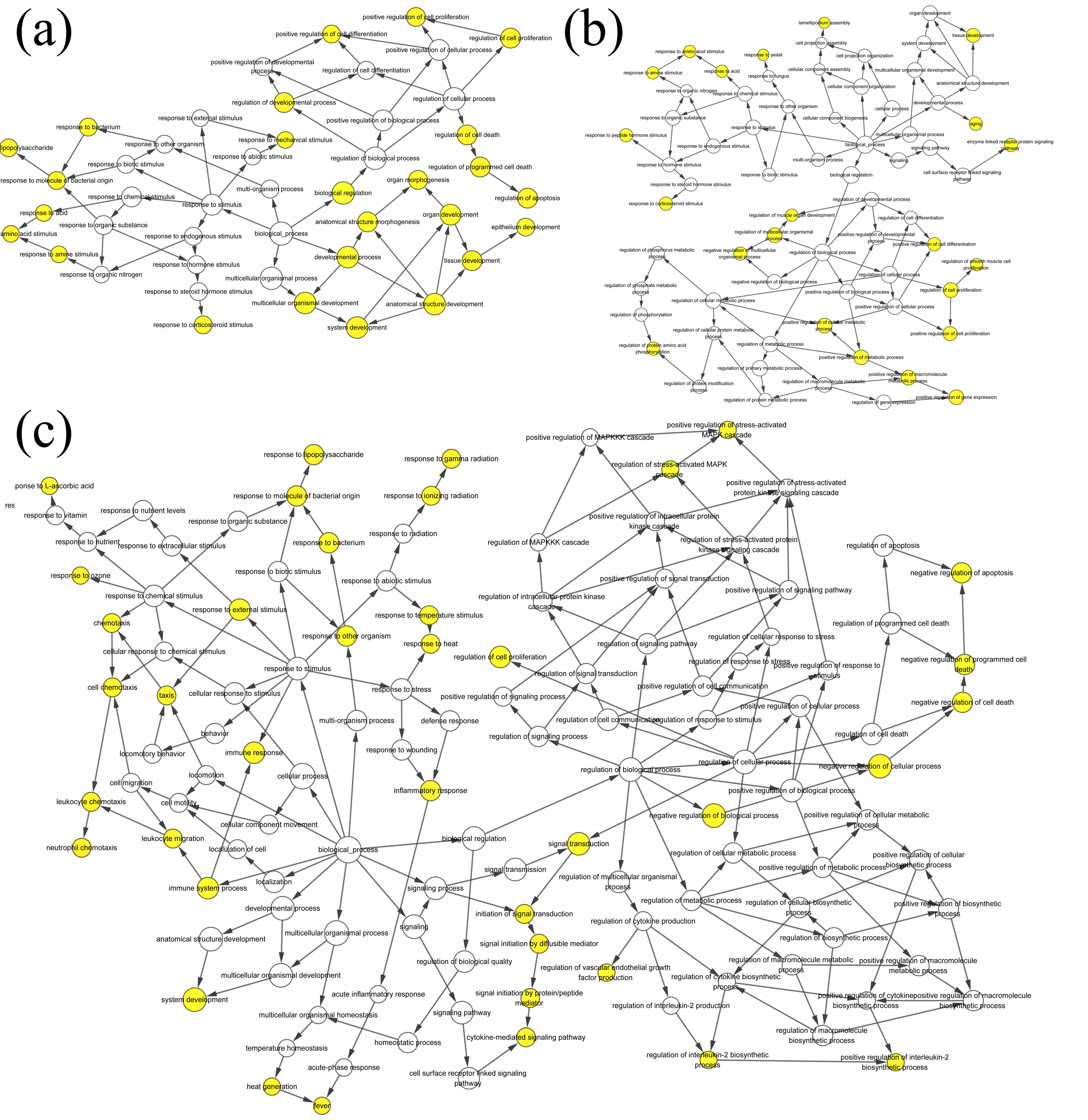

Supplement: Supplementary file 10 [file Image8.TIF]

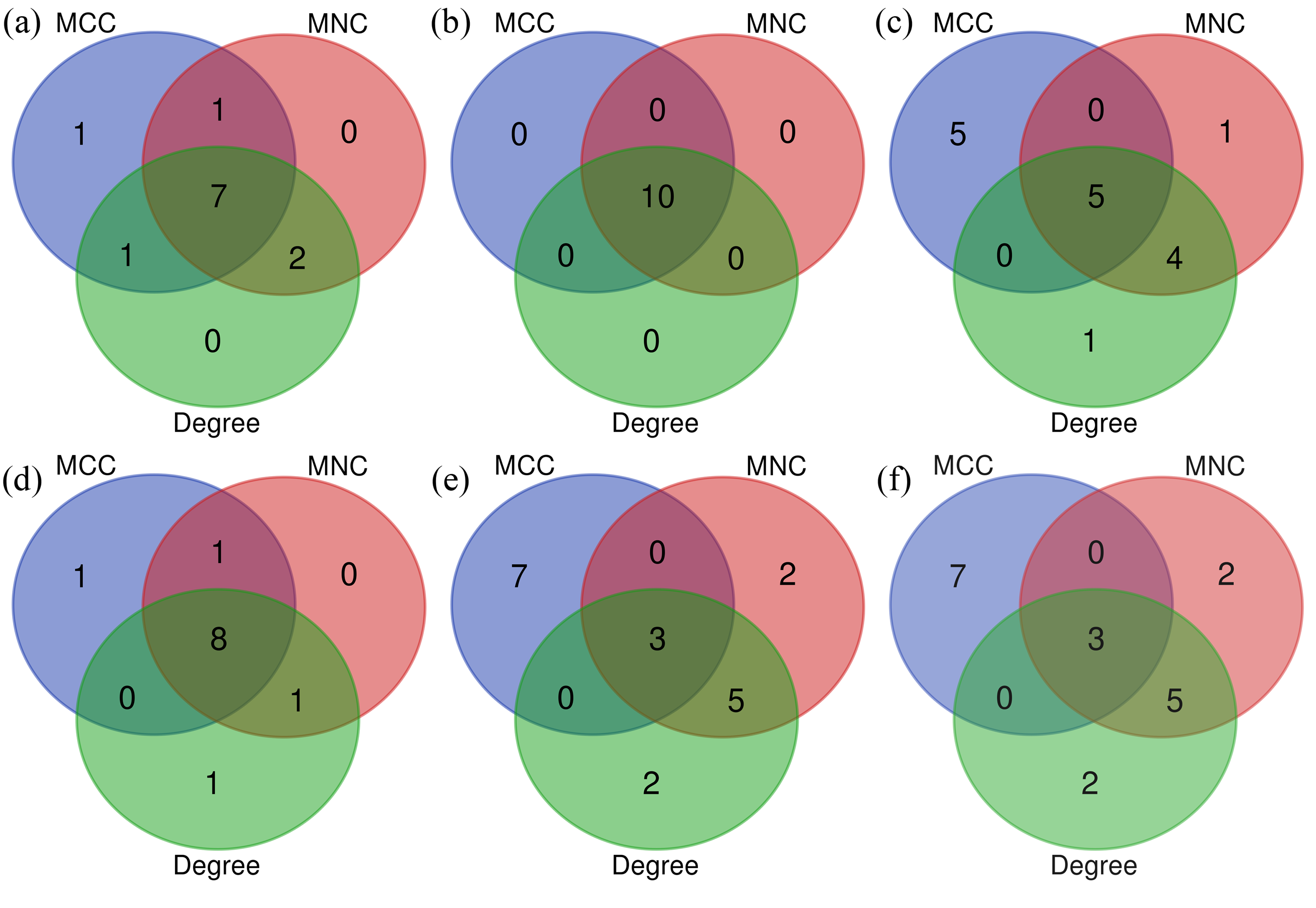

Supplement: Supplementary file 11 [file Image7.TIFF]
