# Supplementary material for: Transcriptome Sequencing Reveals Key Genes in Three Early Phases of Osteogenic, Adipogenic, and Chondrogenic Differentiation of Bone Marrow Mesenchymal Stem Cells in Rats
Source: Front Mol Biosci. 2022 Feb 11;8:782054. doi: 10.3389/fmolb.2021.782054 (PMC8873985; doi:10.3389/fmolb.2021.782054)
Supplement: Supplementary file 4 [file Table1.docx]

| **Table 1.** List of the top 10 hub genes selected by degree methods in cytoHubba | |
| --- | --- |
| Comparisons | Hub genes |
| OS3h *vs.* BMSCs | Ccna2, Cdc20, Il6, Mad2l1, Top2a, Kif11, Mcm4, Asf1b, Rrm1, Pa2g4 |
| OS12h *vs.* BMSCs | Il6, Mad2l1, Top2a, Ccna2, Cdc20, Mcm6, Mcm3, Kif11, Mcm4, Cdc6 |
| OS72h *vs.* BMSCs | Il6, Cd44, Il1b, Ccl2, Kif11, Notch1, Asf1b, Cdc20, Mcm3, Pbk |
| AD3h *vs.* BMSCs | Il6, Ccna2, Cdc20, Kif11, Top2a, Mad2l1, Gsk3b, Mcm6, Asf1b, Mcm3 |
| AD12h *vs.* BMSCs | Ccna2, Mad2l1, Mcm3, Top2a, Mcm6, Kif11, Cdc20, Rrm1, Mcm4, Asf1b |
| AD72h *vs.* BMSCs | Il6, Notch1, Igf1, Il1b, Kif11, Cdc20, Asf1b, Kif20a, Top2a, Rhoc |
| CH3h *vs.* BMSCs | Il6, Mad2l1, Top2a, Cdc20, Kif11, Ccna2, Mcm3, Sspo, Mcm6, Mcm4 |
| CH12h *vs.* BMSCs | Il6, Jun, Notch1, Lep, Mmp2, Acta1, Pik3r1, Csf2, Ldlr, Gja1 |
| CH72h *vs.* BMSCs | Il6, Rac1, Il1b, Acta1, Ccl2, Vcl, Ctnna1, Sox9, Actn2, Actg2 |
| OS3h *vs.* AD3h | Il6, Jun, Notch1, Pik3r1, Sox9, Vcl, Ctgf, Nck1, Rhoq, Gnai1 |
| OS3h vs. CH3h | Il6, Jun, Notch1, Sox9, Vcl, Rnd1, Ccl2, Ctgf, Notch2, Lpl |
| CH3h *vs.*AD3h | Jun, Csf2, Sox9, Ccl2, Rasa1, Il1b, Epha2, Cxcl1, Il1a, Rnd1 |
| OS, osteogenic differentiation; CH, chondrogenic differentiation AD, adipogenic differentiation | |
